# Identification of behaviour change components in swallowing interventions for head and neck cancer patients: protocol for a systematic review

**DOI:** 10.1186/s13643-015-0077-4

**Published:** 2015-06-20

**Authors:** Roganie Govender, Christina H Smith, Stuart A Taylor, Daphne Grey, Jane Wardle, Benjamin Gardner

**Affiliations:** Head and Neck Cancer Centre, University College London Hospital and Health Behaviour Research Centre, University College London, 250 Euston Road, London, NW1 2PQ UK; Division of Psychology and Language Sciences, University College London, London, UK; Centre for Medical Imaging, University College London, London, UK; Bloomsbury Healthcare Library, London, UK; Health Behaviour Research Centre, University College London, London, UK; Department of Psychology, Institute of Psychiatry, Psychology and Neuroscience (IoPPN), Kings College London, London, UK; UCL Centre for Behaviour Change, University College London, London, UK

**Keywords:** Head and neck cancer, Dysphagia, Swallowing rehabilitation, Behaviour change, Behavioural interventions, Systematic review

## Abstract

**Background:**

Dysphagia (difficulty in swallowing) is a predictable consequence of head and neck cancer and its treatment. Loss of the ability to eat and drink normally has a devastating impact on quality of life for survivors of this type of cancer. Most rehabilitation programmes involve behavioural interventions that include swallowing exercises to help improve swallowing function. Such interventions are complex; consisting of multiple components that may influence outcomes. These interventions usually require patient adherence to recommended behaviour change advice. To date, reviews of this literature have explored whether variation in effectiveness can be attributed to the type of swallowing exercise, the use of devices to facilitate use of swallowing muscles, and the timing (before, during or after cancer treatment). This systematic review will use a behavioural science lens to examine the content of previous interventions in this field. It aims to identify (a) which behaviour change components are present, and (b) the frequency with which they occur in interventions deemed to be effective and non-effective.

**Methods/design:**

Clinical trials of behavioural interventions to improve swallowing outcomes in patients with head and neck cancers will be identified via a systematic and comprehensive search of relevant electronic health databases, trial registers, systematic review databases and Web of Science. To ascertain behaviour change intervention components, we will code the content for its theory basis, intervention functions and specific behaviour change techniques, using validated tools: the Theory Coding Scheme, Behaviour Change Wheel and Behaviour Change Technique Taxonomy v1. Study quality will be assessed for descriptive purposes only. Given the specialisation and focus of this review, a small yield of studies with heterogeneous outcome measures is anticipated. Therefore, narrative synthesis is considered more appropriate than meta-analysis. We will also compare the frequency of behavioural components in effective versus non-effective interventions, where effectiveness is indicated by statistically significant changes in swallowing outcomes.

**Discussion:**

This review will provide a synthesis of the behaviour change components in studies that currently represent best evidence for behavioural swallowing interventions for head and neck cancer patients. Results will provide some guidance on the choice of optimal behavioural strategies for the development of future interventions.

**Systematic review registration:**

PROSPERO CRD42015017048

**Electronic supplementary material:**

The online version of this article (doi:10.1186/s13643-015-0077-4) contains supplementary material, which is available to authorized users.

## Background

Head and neck cancer is a cluster term that refers to cancers that arise in the oral cavity, pharynx, larynx, paranasal sinuses, nasal cavity or salivary glands. Since the 1990s, trends have suggested a 30 % increase for oral cancer and a 50 % increase for oropharyngeal cancer [1]. Risk factors for the increase include oral human papillomavirus (HPV) infection and betel nut chewing, as much as the more commonly reported smoking- and alcohol-related causality. The age at which individuals develop head and neck cancer has dropped, meaning that many are still actively employed. Cancer Research UK [2] have estimated the current lifetime risk for a newborn infant of developing head and neck cancer is 1 in 84 for males and 1 in 160 for females. Advances in treatment have improved 5-year survival rates, but this has resulted in a corresponding increase in functional burden such as swallowing difficulties. There are therefore a greater number of individuals, often still of employment age, living longer following their cancer treatment, but with significant functional morbidity. The need to optimise interventions to reduce this morbidity has become increasingly important.

Dysphagia (difficulty in swallowing) is a highly prevalent morbidity following oncological treatment for head and neck cancers, affecting most patients at some stage over the course of treatment [3, 4]. The presence of a tumour in the mouth or throat may result in problems with eating and drinking, but the treatments for cancer (surgery, radiotherapy, chemotherapy) also cause alterations to swallowing function which may persist for many months or even years [5, 6]. Some individuals never regain the ability to eat and drink normally following treatment. Surgery may involve the removal of important oropharyngeal or laryngeal structures with resultant changes to the anatomy and physiology for normal swallowing. The side effects of radiotherapy include a dry mouth, taste changes, fibrosis and stiffening of tissues, which all affect the movement of this finely tuned dynamic process. Dysphagia is also a known late-effect of radiotherapy, meaning that new swallowing symptoms may arise years after the treatment is completed [7]. Difficulty swallowing is often rated as the most significant factor affecting quality of life amongst survivors of head and neck cancers [8, 9]. It has also been identified by head and neck cancer patients, as one of the highest priorities for rehabilitation [10].

### Description of the condition as related to the target population

Individuals who are diagnosed with head and neck cancer may experience some changes to their swallowing function as one of the early symptoms that prompt their visit to a doctor. Usually, most patients continue to maintain an oral diet at this stage. However, the treatments for this type of cancer are known to have a significant impact on the physiology of normal swallow function [3, 4]. Most notably, problems may be associated with swallow safety (aspiration of food and drink into the lungs) or swallow efficiency (prompt and timely transit of food and drink from the mouth through to the oesophagus with complete clearance). The result is that patients are often left with poorer swallowing function *after* treatment. Wall et.al found a prevalence of over 75 % impairment in key swallowing structures across the included studies following chemoradiation [4]. Likewise, surgical interventions may require the complete excision of important structures responsible for airway protection against aspiration, or may result in nerve damage that may affect the timing and co-ordination required for normal swallowing. Reviews on this topic [11–13] suggest that there is value in behavioural interventions such as swallowing exercises in improving the post-treatment function of these patients.

### Description of the intervention and how it may work

In this paper, the term behavioural interventions will include reference to swallowing exercises and strategies, use of a device as part of swallowing exercise and/or specific diet texture instructions. They are defined by the need for patients to perform the recommended behaviour on a regular basis—daily or several times a day. Interventions may be introduced before, during or after oncological treatment.

Swallowing exercises aim to improve muscle strength and range of motion, and consequently muscle function. During oncological treatment, patients may become deconditioned and suffer muscle atrophy due to the less frequent use of the swallowing musculature. They may be required to remain nil by mouth for a time while recovering from surgery, or they may be encumbered by pain, mucositis and other side effects during radiotherapy reducing their oral intake of food and drink. Performing swallowing exercises may maintain the functioning of the oropharyngeal musculature thereby facilitating better recovery of function and possibly preventing the development of fibrotic changes in the muscles [14].

### What is the current evidence for behavioural swallowing interventions in head and neck cancer patients?

Recent systematic reviews have been primarily concerned with identifying the type of intervention and its impact on swallowing outcomes [11], or establishing the methodological quality of previous trials [15]. A review by Cousins et al. [11] found some evidence in support of interventions targeting swallowing and jaw mobility after head and neck cancer, but heterogeneity in outcomes and interventions meant that meta-analysis was not possible. This review highlighted that evidence is limited, and the authors recommended larger, high-quality studies with multiple outcome measures to represent both patient-reported and objective outcomes. However, the review stopped short of a detailed analysis of the intervention descriptions (what happens in the intervention), identifying the key content only as swallowing exercises, electrical stimulation, use of a mechanical jaw stretch device or combinations thereof. The Carnaby and Madhavan review [15] focused on the methodological quality of randomised trials of behavioural interventions in the field of dysphagia rehabilitation. Forty percent of studies in this review were specifically relevant to the head and neck cancer population, but there was no specific information extracted on what makes the interventions effective. An ongoing review by Perry et al. [16] focuses on the effect of swallowing exercises on oral swallowing, aspiration and other related adverse events in patients with advanced head and neck cancers. Based on their published protocol [16], we expect to examine a similar body of evidence to Perry et al. While the Perry et al. review has the specific purpose of examining direct swallowing exercises (e.g. type, dose, frequency) in randomised trials using Cochrane methodology, our review will look more broadly at clinical trials (randomised and non-randomised) with a focus on the behavioural techniques used in these interventions. We believe that these reviews will complement each other offering a broader and more balanced picture of the current evidence in this field.

For behavioural swallowing interventions to impact swallow function, we need *behaviour change* to occur: we can only determine the outcome of an exercise intervention if we are confident that the exercises have been performed as prescribed. Behaviour change cannot be assumed; many behaviour change interventions may fail, not because the intervention is ineffective in modifying the clinical outcome, but because the individual fails to adhere to the recommended advice. This phenomenon has been recognised in the swallowing rehabilitation literature; a previous retrospective study of 497 patients reported a statistically significant difference in functional swallowing status (return to full oral diet) in patients who adhered to their exercises compared with those who did not [14]. Within the clinical context of patient care, we need to ensure that optimised behaviour change techniques are part of our intervention design in order to maximise the chance that the patients are carrying out the recommended advice and behaviours. While findings from previous and ongoing reviews will undoubtedly contribute to knowledge, understanding and clinical choices, a more complete analysis of behavioural swallowing intervention effectiveness also requires assessment of the potential contribution of the behaviour change strategies used. Recent advances in behavioural science have been found to be useful in unpacking the components of complex interventions aimed at changing people’s behaviour. This approach has been applied in other domains such as diet and physical activity [17, 18]. Its application to the field of swallowing rehabilitation is novel.

### Why is it important to do this review?

Over the last decade, great progress has been made in our understanding of swallowing physiology, the impact of oncological treatments on swallow physiology, and patient-reported functional outcomes and quality of life. As noted from the reviews already cited, evidence is accumulating for behavioural interventions that aim to improve the swallowing outcomes for this target population. However, it is clear that the evidence is weakened by the lack of high-quality intervention studies. Any discipline can be advanced by the adoption of progressive and transposable methods developed in other disciplines. Guidance on complex intervention research designs [19], as well as improvements in characterising complex interventions using comprehensive frameworks [20, 21], are examples of this. They take account of the fact that several interacting components of an intervention may impact the outcomes.

We therefore ask the question: Which behaviour change components are reported in swallowing interventions for head and neck cancer patients, and how frequently do they occur in interventions deemed to be effective? This review will examine and characterise the behaviour change components that have been reported in previous swallowing intervention studies with the view to informing the development of new interventions in this field. A clear description of these components will be useful in transparently describing the content of this complex intervention. Specifying intervention content in a consistent way is desirable for replication of effective interventions, and avoiding replication of ineffective interventions. This in turn enhances the ability of the field to accrue evidence that will allow greater confidence in answering questions about what works. It is unlikely at this stage, that we will be able to be conclusive about which behavioural components are most effective, but we can map how frequently they occur in interventions reported to be effective or non-effective.

In this review, we will make use of the Behaviour Change Wheel (BCW) [21], a comprehensive, theoretically based approach to support the design, evaluation and refinement of behavioural interventions. The BCW encompasses a model of behaviour as well as function and policy categories. The model describes nine intervention functions (Education, Training, Persuasion, Coercion, Restriction, Modelling, Enablement, Incentivisation and Environmental Restructuring), and seven policy categories that include service provision and guidelines. It takes account of the broad range of factors that influence behaviour change interventions and is supported by a taxonomy of 93 behaviour change techniques [22] to assist in the identification of the ‘active ingredients’ present in interventions. Behaviour change techniques (BCTs) are defined as *observable and replicable components of behaviour change interventions* [23] that represent the proposed active ingredients of an intervention. The BCT taxonomy provides a standardised and coherent terminology to aid description and identification of BCTs, which are often inconsistently reported [24]. We plan to identify the intervention functions and BCTs employed in the selected studies for this review. It has been suggested that interventions that are explicitly theory based are more effective, but a recent review [25] has highlighted that the majority of health interventions show no link to theory. We will therefore note whether any theory is mentioned in the abstract, introduction or method, using the Theory Coding Scheme (TCS) [26]. To our knowledge, no reviews within the field of dysphagia rehabilitation have characterised interventions using this approach. The BCW, the BCT taxonomy and the TCS are validated tools that can be applied retrospectively to descriptions of interventions. In this review, they will provide a toolkit offering structure and coherence to the processes of extraction and synthesis of intervention components.

### Aim

In this review, we aim to:

- Identify the behavioural intervention components reported in the published literature of swallowing interventions for patients with head and neck cancer.
- Examine how frequently these behavioural components occur in interventions deemed to be effective vs non-effective.

## Methods/design

We have consulted the PRISMA-P guidelines [27] in preparing this protocol.

### Criteria for including studies in this review

#### Types of studies

Only peer-reviewed studies published in English will be included. Randomised and non-randomised studies will be included provided that an intervention group and a suitable comparator/control group is part of the study design. We will not apply any date restrictions or minimum sample size.

#### Types of participants/population group

Individuals over the age of 18, diagnosed with head and neck cancer (excluding brain) and having/had treatment via one of the main modalities of surgery, radiotherapy, chemoradiation or combinations thereof.

#### Types of intervention/s

In this review, ‘behavioural intervention’ makes reference to swallowing exercises, instructions to adhere to specific diet texture recommendations and swallowing strategies: instructions for swallowing compensations and manoeuvres. It requires the patient to perform a particular behaviour on a regular basis. Interventions that include a device (for example, therabite, theraspoons, spatulas) as part of an exercise package will also be included provided the device is part of a behaviour change programme to improve swallowing outcomes. Interventions designed solely to treat trismus or improve mouth opening will be excluded if no swallowing outcome is assessed. Medical, surgical, prosthetic, pharmacological and neuromuscular or electrical stimulation type interventions will be excluded.

#### Type of comparator group

The comparison group may be an active or inactive control. For review purposes, an active control refers to a group that is still given some intervention (usual care or sham exercises) rather than no intervention at all. It is recognised that in the clinical context of cancer treatment, it may be ethically inappropriate to have a parallel group with no treatment. Therefore, standard treatment, usual care at the time of the study, or usual care and a sham exercise group will be acceptable as controls.

### Types of outcome measures

The main outcome of interest is swallowing function. At least one measure of swallowing must therefore be reported as an outcome. These measures usually fall into three categories: objective or instrumental measures, clinician-rated measures and patient-rated measures.

### Identification of studies

#### Information sources

The following electronic health databases will be searched: MEDLINE, CINAHL, Embase, AMED, PsychInfo and the Cochrane Library including CENTRAL. Additional searches will be carried out on Google Scholar, Web of science and the meta-registries of Trials Databases (ClinicalTrials.gov and ISRCTN). We will also search the WHO International Clinical Trials Registry Platform (ICTRP) and the Australian New Zealand Clinical Trials Register (ANZCTR). In addition, we will hand-search the reference lists of any directly relevant systematic reviews as well as the included articles for any additional studies.

#### Search strategy

A search strategy to identify relevant studies will be developed in conjunction with a subject librarian. We have identified initial terms from other relevant reviews and from MeSH headings of key articles from an initial scoping exercise. We will use the terms deglutition OR swallow* OR Dysphagi* in combination with the exploded terms for ‘head and neck neoplasms.’ We will also use the terms therap* OR rehabilitation OR exercise OR behav* OR ‘swallowing training.’ The search will be focused to capture the most relevant reports by limiting to clinical trials and reviews, and excluding oesoph* and brain neoplasms. We will limit the search to English language but not apply a date limitation. An example of the search strategy used in MEDLINE is illustrated in Table 1.

**Table 1:** Example of a search strategy for MEDLINE

Search history
Evidence Services | library.nhs.uk
1. MEDLINE; exp DEGLUTITION/OR exp DEGLUTITION DISORDERS/; 47,421 results.
2. MEDLINE; (deglutition OR swallow* OR dysphagi*).ti,ab; 36,529 results.
3. MEDLINE; exp HEAD AND NECK NEOPLASMS/; 246,253 results.
4. MEDLINE; (‘head and neck’ OR otorhinolaryng* OR ‘head neck’ OR ‘head-neck’ OR oral OR oropharyn* OR hypopharyn* OR laryn* OR nasopharyn* OR pharyn* OR throat OR mouth OR HNSCC OR SCCHN).ti,ab; 658,144 results.
5. MEDLINE; (cancer* OR carcinoma* OR neoplas* OR tumor* OR tumour* OR malignan* OR SCCA).ti,ab; 2,319,509 results.
6. MEDLINE; exp REHABILITATION/; 153,977 results.
7. MEDLINE; exp BEHAVIOR/; 1,232,359 results.
8. MEDLINE; (therap* OR rehabilitation OR exercise* OR behav* OR "swallowing training").ti,ab; 2,913,323 results. 9. MEDLINE; 1 OR 2; 67,740 results.
10. MEDLINE; 4 AND 5; 143,824 results.
11. MEDLINE; 3 OR 10; 310,277 results.
12. MEDLINE; 6 OR 7 OR 8; 3,852,180 results.
13. MEDLINE; 9 AND 11 AND 12; 3060 results.
14. MEDLINE; 13 [Limit to: (Publication Types Clinical Trial, All or Systematic Reviews)]; 258 results.
15. MEDLINE; (esophag* OR oesophag* OR brain).ti; 345,597 results.
16. MEDLINE; *ESOPHAGEAL NEOPLASMS/; 32,758 results.
17. MEDLINE; 15 OR 16; 349,564 results.
18. MEDLINE; 14 not 17 [Limit to: (Publication Types Clinical Trial, All or Systematic Reviews)]; 151 results.
19. MEDLINE; 18 [Limit to: English Language and (Publication Types Clinical Trial, All or Systematic Reviews)]; 140 results.
20. MEDLINE; (rehabilitation OR exercise* OR behav* OR "swallowing training").ti,ab; 1,125,728 results.
21. MEDLINE; exp EXERCISE THERAPY/; 31,541 results.
22. MEDLINE; exp BEHAVIOR/ OR exp REHABILITATION/; 1,355,478 results.
23. MEDLINE; 20 OR 21 OR 22; 2,136,331 results.
24. MEDLINE; 9 AND 11 AND 23; 1096 results.
25. MEDLINE; 24 [Limit to: English Language and (Publication Types Clinical Trial, All or Systematic Reviews)]; 101 results.
26. MEDLINE; 25 not 17 [Limit to: English Language and (Publication Types Clinical Trial, All or Systematic Reviews)]; 90 results.

### Study records

#### Data management and selection

Articles from all searches will be combined and duplicates removed. Titles and abstracts will be screened against eligibility criteria by two members of the review team (RG and DG), a specialist head and neck speech and language therapist and a subject librarian. The full-text versions of studies deemed eligible by either team member will be obtained. The articles will be imported into Mendeley Web (bibliographic database) for easy access amongst the review team members. Multiple reports of the same intervention study will be grouped together for data extraction. Studies will be assessed for eligibility using a pre-agreed template form. The form will specify the eligibility criteria and consist of a table with a complete list of all the full-text studies retrieved. Two reviewers with expertise in dysphagia (RG, CS) will independently select one of three categories (include, exclude, unsure) for each study. Repeat articles relating to the same study sample will be grouped together. Reasons will be recorded for studies that are excluded. Uncertainties and discrepancies will be resolved through discussion. A third member of the research team (BG) will be available to assist in resolving any disagreements. The final list of studies to be included in the review will be imported into NVIVO 10 (QSR International), a relational database for organising and analysing qualitative data. A PRISMA flowchart will be completed to summarise this process.

### Data extraction

A pre-agreed and piloted data extraction form will be used to collect the relevant information from the selected studies. This will include study characteristics such as type of study, participants, length of follow-up, outcomes and quality assessment. Intervention characteristics will include information about the target behaviours, theory basis, intervention functions, BCTs as well as fidelity in delivering the intervention. Data will be extracted from all studies by one member of the team (RG). Two other members of the team (CS or BG) will independently extract data for a minimum of 25 % of studies randomly selected using a random list generator (https://www.random.org/). Independent coding of study characteristics will be done by CS, and intervention characteristics by BG. Two review authors (RG and BG) are trained in the use of the Behaviour Change Technique Taxonomy v1 [22] and will code studies using this framework. To ensure consistency in interpreting the framework, the two reviewers will independently code a small number of studies from those not selected, before coding the selected studies. Uncertainties can then be discussed prior to the coding of the studies included in the review. Inter-rater agreement will be calculated for the study characteristics as a whole and for each of the intervention characteristic (target behaviour, intervention function, BCT) independently. This will be computed using the kappa measure [28] and interpreted according to the classification proposed by Landis and Koch [29]. For the BCT coding, we will adopt a conservative approach to calculating agreement based on the BCTs judged to be present by either of the two coders. We will however also report kappa scores for all 93 BCTs with recognition that these scores may be inflated due to agreement on the absence of most of the 93 BCTs.

#### Quality assessment

As part of the data collection process, we will use the 11-item scale described by van Tulder [30] to assess the methodological quality of the selected studies. This information will be used to provide a summary of the quality ratings of the studies included, for descriptive purposes only. We have expanded on our reasons for this choice in the ‘Discussion’ section.

### Data synthesis

#### Quantitative synthesis

Based on other similar reviews [11, 15], we anticipate variation in outcomes, instruments used to derive outcomes and other aspects of intervention content and delivery. Calculating and pooling effect sizes is therefore untenable. It is also anticipated that outcomes may be reported at multiple timepoints such as 3, 6, 12 months post-treatment. We will use the most commonly reported timepoint (e.g. 6 months) to derive statistical significance of change in outcome, but if necessary, we will report effects at multiple timepoints. Swallowing outcomes will be broadly categorised into patient-reported outcomes, clinician-rated outcomes and outcomes derived from instrumental and/or other objective measures. Where multiple outcome measures in the same study vary with regard to effectiveness, the primary swallowing outcome will take precedence. A possible template table (Additional file 1: Table S1) provides a visual representation of how we may report these findings. The presence of specific behavioural components across studies will be tabulated and frequency and ratio measures computed. This information will allow the reader to tell at a glance how many intervention components have been used more frequently in effective than ineffective interventions, and exactly how much more frequently. We will provide further information on the table by indicating the types of outcome measures reported. The studies will also be ordered according to their quality rating summary score. We may also tabulate the full quality assessment of individual studies for descriptive purposes.

#### Narrative synthesis

We have consulted the guidance on conducting a narrative synthesis described by Popay and colleagues [31] and anticipate the use of their general framework that describes four main elements:

*Developing a theory of how the intervention works, why and for whom*

*Developing a preliminary synthesis of findings of included studies*

*Exploring relationships in the data*

*Assessing the robustness of the synthesis (p11)*

This framework (we anticipate structuring the synthesis using the latter three elements) will inform our synthesis and discussion of findings. In addition, we will provide a perspective on the clinical and research implications of this review.

## Discussion

This review will offer a novel method for characterising behavioural interventions to improve swallowing function using established frameworks from the discipline of behavioural science. Previous reviews have not deconstructed the intervention processes, so while they provide a valuable summary of the available evidence, there is a significant gap in terms of providing information on what happens and why. Consequently, we remain uncertain about how best to improve the design of behavioural interventions to facilitate better outcomes. In this review, we will provide a synthesis of the ‘active ingredients’ or proposed mechanisms of behaviour change described in the swallowing intervention studies that currently represent best available evidence for the population of interest. We will also endeavour to comment on the use of theory in informing our current interventions. As this approach is new in the field, it is not our intention to be conclusive about which BCTs are most effective. We do however wish to note how frequently they are reported in interventions deemed effective vs non-effective. This mapping of information will be useful in aiding the selection of content for future swallowing interventions. While it will not be possible to conclude that the BCT itself is responsible for effects, any such observed covariance of intervention components with effectiveness will help point intervention designers in the right direction.

We will report on the quality of the included studies, but as we do not anticipate any pooling of data or meta-analysis, studies with poorer quality ratings will be retained. Nonetheless, we see value in performing a quality assessment: we believe that a summary of study quality will provide a useful snapshot of the methodological rigour of previous studies in this field and expands upon previous work that aimed to describe the quality of clinical trials in swallowing rehabilitation [15]. For ease of comparison, we have chosen to use the same quality assessment tool used in the review by Carnaby and Madhavan [15]. Discussion about quality assessment will be integrated into our narrative synthesis. It will help contextualise findings and as suggested by Popay and colleagues is also helpful in assessing the robustness of the synthesis [31].

This review is the first attempt to apply the behaviour change framework and taxonomy to the literature on swallowing rehabilitation interventions. We anticipate that descriptions of interventions are likely to lack detail but have decided against contacting authors for further information. We have instead decided to code these interventions based solely on the information which authors have made publically available including appendices and published intervention manuals. While we are aware of the need to distinguish between reporting and conduct of the intervention, we also believe that consumers of research are usually only in a position to interpret studies based on the published report. The data extraction will therefore also be based on information available to all readers. We anticipate that the findings could be useful in promoting better reporting of future studies. Better specified interventions are more easily replicated and while it is not an explicit intention of the review, raising awareness of this may be a welcome influence on intervention designers.

This review adopts a new framework that we hope will help clinicians identify and begin to understand the behaviour change components of the complex therapy interventions they provide to their patients. We are presented with new challenges in the quest to assimilate the evidence for interventions. As articulated by Petticrew [32], it may be necessary to expand our enquiry from ‘what works’ when swallowing interventions are delivered to ‘what happens’ that might make them work. We are required to explore a broader question to ascertain *what* has happened in previous interventions, as opposed to focusing solely on intervention effects. For a field which is relatively new to evidence synthesis and with relatively few high-quality randomised studies, this approach offers a way of systematically gathering the available evidence as a first step to developing hypotheses for further testing.

### Reporting and dissemination of findings

We plan to report this review in accordance with the PRISMA guidelines [33] and to publish the findings in a peer-reviewed journal. We also expect to present these findings at relevant national and international scientific meetings.

## Additional file

Additional file 1: Table S1. Frequency of BCTs in interventions reported to be effective or ineffective at 6-month follow-up.

## Abbreviations

BCT: behaviour change technique/s
BCW: Behaviour Change Wheel
HEE: Health Education England
ISRCTN: International Standard Randomized Controlled Trial Number
MeSH: Medical Subject Headings
NHS: National Health Service
NIHR: National Institute of Health Research
PRISMA: Preferred Reporting of Items for Systematic Reviews and Meta Analyses
