# Supplementary material for: Identification of behaviour change components in swallowing interventions for head and neck cancer patients: protocol for a systematic review
Source: Syst Rev. 2015 Jun 20;4:89. doi: 10.1186/s13643-015-0077-4 (PMC4474547; doi:10.1186/s13643-015-0077-4)
Supplement: Additional file 1: Table S1. — Frequency of BCTs in interventions reported to be effective or ineffective at 6-month follow-up. [file 13643_2015_77_MOESM1_ESM.docx]

| Study (grouped by effectiveness, as judged by statistical significance) | Quality  rating | Behaviour Change Techniques | | | | |  |  |  |
| --- | --- | --- | --- | --- | --- | --- | --- | --- | --- |
|  |  | Goal setting behaviour | Self monitoring of behaviour | Instruction on how to perform behaviour | Social support  (practical) | Information about health consequences | Demonstration of behaviour | Prompts and cues | Credible source |
| **Effective** |  |  |  |  |  |  |  |  |  |
| Study 1 | 8 | P C | P C | PC |  |  | P C |  | PC |
| Study 2 | 7 |  | P O |  |  | P O |  | P O |  |
| Study 3 | 7 | P C 0 | P C O | P C O |  | P C O | P C O |  |  |
| Study 4 | 5 |  |  | C O |  |  | C O |  | C O |
| Study 5 | 4 |  |  | P |  |  | P |  | P |
| TOTAL |  | 2 | 3 | 4 | 0 | 2 | 4 | 1 | 3 |
|  |  |  |  |  |  |  |  |  |  |
| **Non Effective** |  |  |  |  |  |  |  |  |  |
|  |  |  |  |  |  |  |  |  |  |
| Study 6 |  |  |  |  |  |  |  |  |  |
| Study 7 |  |  |  |  |  |  |  |  |  |
| Study 8 |  |  |  |  |  |  |  |  |  |
| Study 9 |  |  |  |  |  |  |  |  |  |
| Study 10 |  |  |  |  |  |  |  |  |  |
| TOTAL |  |  |  |  |  |  |  |  |  |

Table S1: Frequency of BCTs in interventions reported to be effective or ineffective at 6-month follow-up.

Note. Cells containing at least one letter indicates the presence of the BCT in the intervention, and the letter indicates the type of outcome measures reported in the study, where P = patient reported measure, C= clinician rated measure, O= measure derived from instrumental/objective assessment. Quality Rating: higher numbers represent better study quality and lower risk of bias.
